# Triggering a switch from basal- to luminal-like breast cancer subtype by the small-molecule diptoindonesin G via induction of GABARAPL1

**DOI:** 10.1038/s41419-020-02878-z

**Published:** 2020-08-15

**Authors:** Minmin Fan, Jingwei Chen, Jian Gao, Wenwen Xue, Yixuan Wang, Wuhao Li, Lin Zhou, Xin Li, Chengfei Jiang, Yang Sun, Xuefeng Wu, Xudong Wu, Huiming Ge, Yan Shen, Qiang Xu

**Affiliations:** 1 State Key Laboratory of Pharmaceutical Biotechnology, School of Life Sciences, Nanjing University, 22 Han Kou Road, Nanjing, 210093 China; 2 Department of Pathology, Nanjing Medical University, 140 Hanzhong Road, Nanjing, 210029 China

**Keywords:** Breast cancer, Pharmacogenetics

## Abstract

Breast cancer is a heterogeneous disease that includes different molecular subtypes. The basal-like subtype has a poor prognosis and a high recurrence rate, whereas the luminal-like subtype confers a more favorable patient prognosis partially due to anti-hormone therapy responsiveness. Here, we demonstrate that diptoindonesin G (Dip G), a natural product, exhibits robust differentiation-inducing activity in basal-like breast cancer cell lines and animal models. Specifically, Dip G treatment caused a partial transcriptome shift from basal to luminal gene expression signatures and prompted sensitization of basal-like breast tumors to tamoxifen therapy. Dip G upregulated the expression of both GABARAPL1 (GABA_A_ receptor-associated protein-like 1) and ERβ. We revealed a previously unappreciated role of GABARAPL1 as a regulator in the specification of breast cancer subtypes that is dependent on ERβ levels. Our findings shed light on new therapeutic opportunities for basal-like breast cancer via a phenotype switch and indicate that Dip G may serve as a leading compound for the therapy of basal-like breast cancer.

## Introduction

Breast cancer is a heterogeneous disease comprised of different molecular subtypes, which can be identified through gene or biomarker expression analyses and are predictive of prognosis^1–3^. Luminal-like breast cancer, which includes luminal A and B subtypes, is characterized by the expression of estrogen receptor (ER) and/or progesterone (PR) and confers a more favorable prognosis partially due to anti-hormone therapy responsiveness^4^. Conversely, basal-like breast cancer, such as ER^-^ PR^-^ HER2^-^ triple negative breast cancer, has the highest recurrence rate and the worst overall survival rate among all the breast cancer subtypes^5^. The paucity of therapeutic targets and the poor disease prognosis have fostered a major effort to develop new treatment approaches for patients with basal-like breast cancer.

The distinguishing features of luminal and basal-like breast cancer cells have been explained by a cell-of-origin hypothesis, i.e., originating from luminal progenitors and breast epithelial stem cells, respectively^6^. However, recent studies have proposed luminal progenitors as the common origin of both luminal and basal-like breast cancers^7–9^. A tumorigenic subpopulation of luminal progenitors could dedifferentiate to acquire a basal-like phenotype. Indeed, basal-like cancer cells show a high degree of heterogeneity and plasticity^10–13^. Nuclear extracts of basal-like cancer cells are reported to be sufficient to induce a luminal-to-basal phenotype switch^11^. Moreover, loss of luminal-defining factors, such as forkhead box transcription factor (FOXA1) or GATA-binding protein 3 (GATA3), causes a shift from luminal to basal gene expression signatures^12–14^. A recent study demonstrated that intervention of platelet-derived growth factor (PDGF)-CC activity in mouse models results in the conversion of basal-like breast cancers into a luminal state that confers sensitivity to endocrine therapy^10^. Therefore, pharmacologic strategies for triggering the switch from basal-like cancer to a luminal subtype would benefit the clinical therapy of basal-like breast cancer patients.

GABARAPL1 (GABA_A_ receptor-associated protein-like 1), a member of the GABARAP family, is highly evolutionarily conserved^15^. Its coding gene was first identified as an early estrogen-induced gene^16^. Apart from its function in autophagy, GABARAPL1 plays a potential role during tumor progression^17,18^. *GABARAPL1* expression is robustly downregulated in breast tumors and those patients with high *GABARAPL1* levels have a low risk of metastasis^19^. GABARAPL1 overexpression inhibits cell proliferation, colony formation and invasion in breast cancers in vitro^19,20^. Decreased *GABARAPL1* expression has also been observed in patients with acute myeloid leukemia (AML)^21^. Interestingly, its expression can be induced during all-*trans* retinoic acid (ATRA)-induced neutrophil differentiation, implying a novel function in cell differentiation. Our previous study revealed that the oligostilbenoid natural product diptoindonesin G (Dip G) induced myeloid differentiation of AML cells to an equivalent or higher extent than ATRA^22^. Herein, we found that Dip G could induce cell differentiation in basal-like breast cancer cells. Gene profiling analyses showed that Dip G treatment caused a partial transcriptome shift from basal to luminal gene expression signatures. Such a phenotype switch conferred sensitivity to tamoxifen therapy in basal-like breast tumors. In addition, our data identified GABARAPL1, whose expression was increased by Dip G in an ERβ-dependent manner, as a functional regulator of the molecular subtype of breast cancer. These findings shed light on new therapeutic opportunities via a phenotype switch for basal-like breast cancer.

## Results

### Dip G induces luminal differentiation in basal-like cancer cells

A previous study reported that Dip G, a resveratrol aneuploid either naturally isolated from the stem bark of tropical plants such as *Hopea chinensis* or totally synthesized, has robust antiproliferative activity in cancer cells^22–24^. In line with these findings, a dose-and time-dependent inhibition of cell growth by Dip G was observed in the basal-like cancer cell lines MDA-MB-231, SUM1315, and MDA-MB-468 using the trypan blue dye exclusion, soft agar colony formation, and bromodeoxyuridine assays (Fig. S1). Pretreatment with various inhibitors, including a pan-caspase inhibitor (z-VAD-FMK), a necroptosis inhibitor (necrostatin-2) and an autophagy inhibitor (chloroquine), failed to reverse the antiproliferative activity of Dip G in MDA-MB-231 cells (Fig. S2). These results help to rule out the possibility that Dip G inhibits the growth of these basal-like cancer cells via induction of caspase-dependent apoptosis, necroptosis or autophagic cell death.

**Fig. 1:**
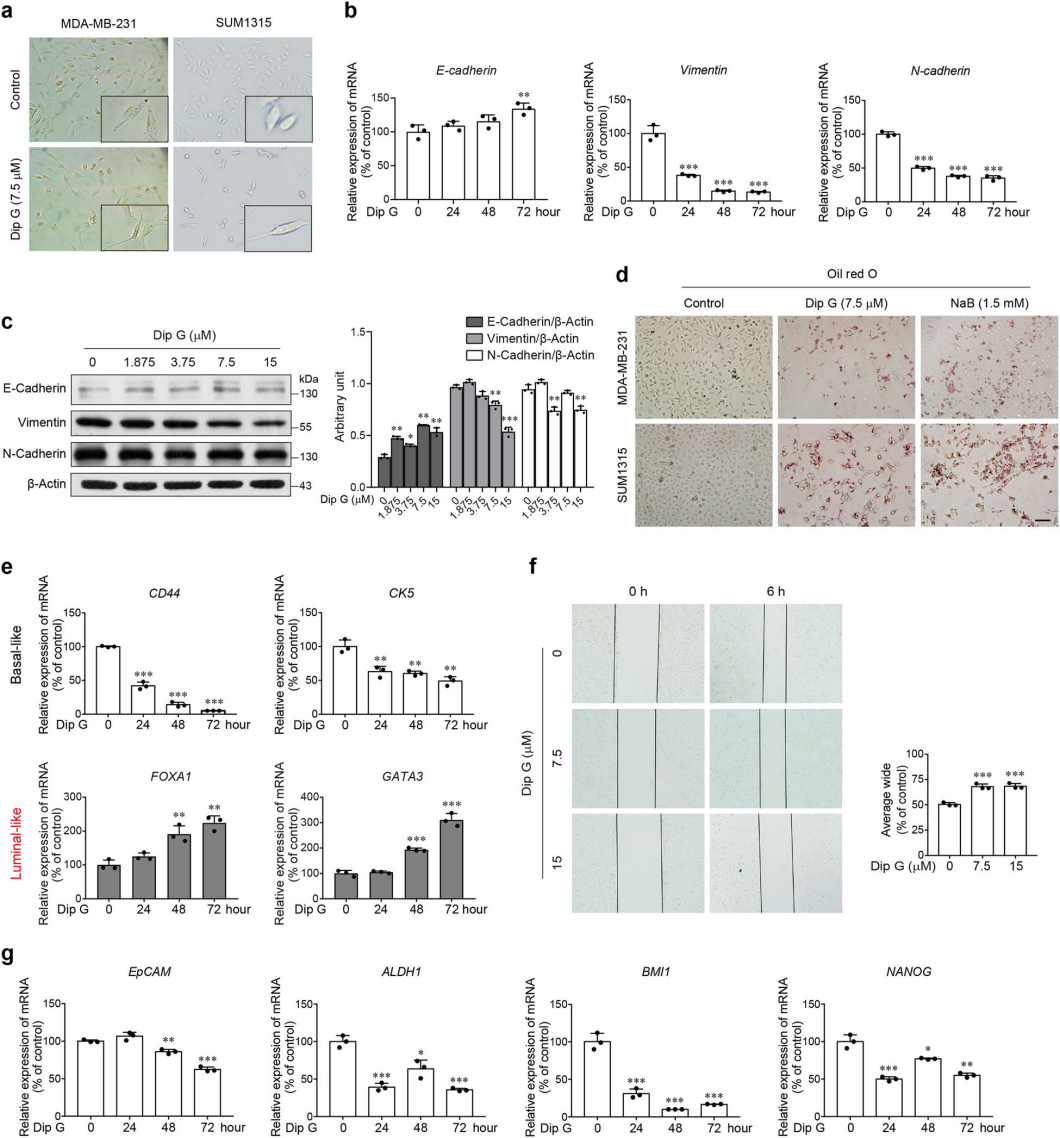
Differentiation-inducing activity of Dip G in basal-like breast cancer cell lines. **a** Cellular morphology in cells treated with Dip G (7.5 μM) for 72 h. Representative images are shown. Zoom-in factor: 4. **b** MDA-MB-231 cells were treated with Dip G (7.5 μM) for the indicated times. The mRNA levels of E-cadherin, vimentin and N-cadherin were determined by real-time RT-PCR. β-Actin was used as an internal control. **c** MDA-MB-231 cells were treated with various concentrations of Dip G for 24 h. The protein levels of E-cadherin, vimentin and N-cadherin were determined by western blot. β-Actin was used as a loading control. The densitometry of the immunoblots was performed with image J software and is presented in the histograms. The data are shown as the mean ± SD of three independent experiments. **d** Cells were treated with Dip G or NaB for 72 h. Neutral fat contents were stained using the Oil Red O staining protocol. Scale bar: 50 μm. **e** Expression of basal *CD44* and *CK5* and luminal *FOXA1* and *GATA3* genes in MDA-MB-231 cells treated with Dip G (7.5 μM) for the indicated times. **f** Wound-healing assay for the assessment of migration in cells treated with various concentrations of Dip G for 6 h. Right panel: Representative photomicrograph. Left panel: Calculated wound-healing areas. **g** Expression of stemness-associated genes *EpCAM*, *ALDH1*, *BMI1*, and *NANOG* in MDA-MB-231 cells treated with Dip G (7.5 μM) for the indicated times. Data are shown as the mean ± S.D. of three independent experiments. *P < 0.05, **P < 0.01, ***P < 0.001 versus the control group without Dip G treatment.

Given the strong correlation between cell growth arrest and the progression of cancer cell differentiation, we assessed cellular morphology and the expression of cell–cell adhesion molecules in Dip G-treated basal-like cancer cells. Both MDA-MB-231 and SUM1315 cells displayed a distinct appearance from the original spindle-shaped morphology after a 72-h treatment with 7.5-μM of Dip G (Fig. 1a). E-cadherin expression was increased, whereas the expression of vimentin and N-cadherin was decreased by Dip G at both the mRNA and protein levels in MDA-MB-231 cells (Fig. 1b, c). Using Oil red O staining to analyze neutral fat contents, we observed a higher intensity of Oil red O in the cells treated with Dip G, which is similar to the result with the cell differentiation inducer NaB (Fig. 1d). Notably, Dip G downregulated the expression of the basal-like genes *CD44* and *cytokeratin (CK) 5*, accompanied by upregulation of the luminal-like genes *FOXA1* and *GATA3* expression in a time-dependent manner (Fig. 1e). The wound-healing assay also showed that Dip G-treated MDA-MB-231 cells were less migratory than the untreated control (Fig. 1f), indicative of the functional property of luminal-like breast cancer cells. Furthermore, considering that CD44^+^ CD24^−^ subpopulation enriched in basal-like breast cancer cells has the stem/progenitor cell properties^25^, we evaluated the effects of Dip G on the stemness. Real-time RT-PCR analysis showed reduced levels of mRNA transcripts of stemness-associated genes, such as *EpCAM*, *ALDH1*, *BMI1*, and *NANOG*, in Dip G-treated MDA-MB-231 cells (Fig. 1g). Accordingly, Dip G reduced the percentage of CD44^+^ CD24^−^ cells and downregulated the canonical Wnt/β-catenin signaling pathway known to regulate the self-renewal and stemness of cancer cells, as evidenced by the increase in the levels of p-β-catenin (Ser33/37/Thr41) and p-β-catenin (Thr41/Ser45) and the decrease in the levels of p-β-catenin (Ser552), p-β-catenin (Ser675) and c-Myc in both MDA-MB-231 and SUM1315 cells (Fig. S3).

### Dip G induces a partial transcriptome shift from basal to luminal gene expression signatures

**Fig. 2:**
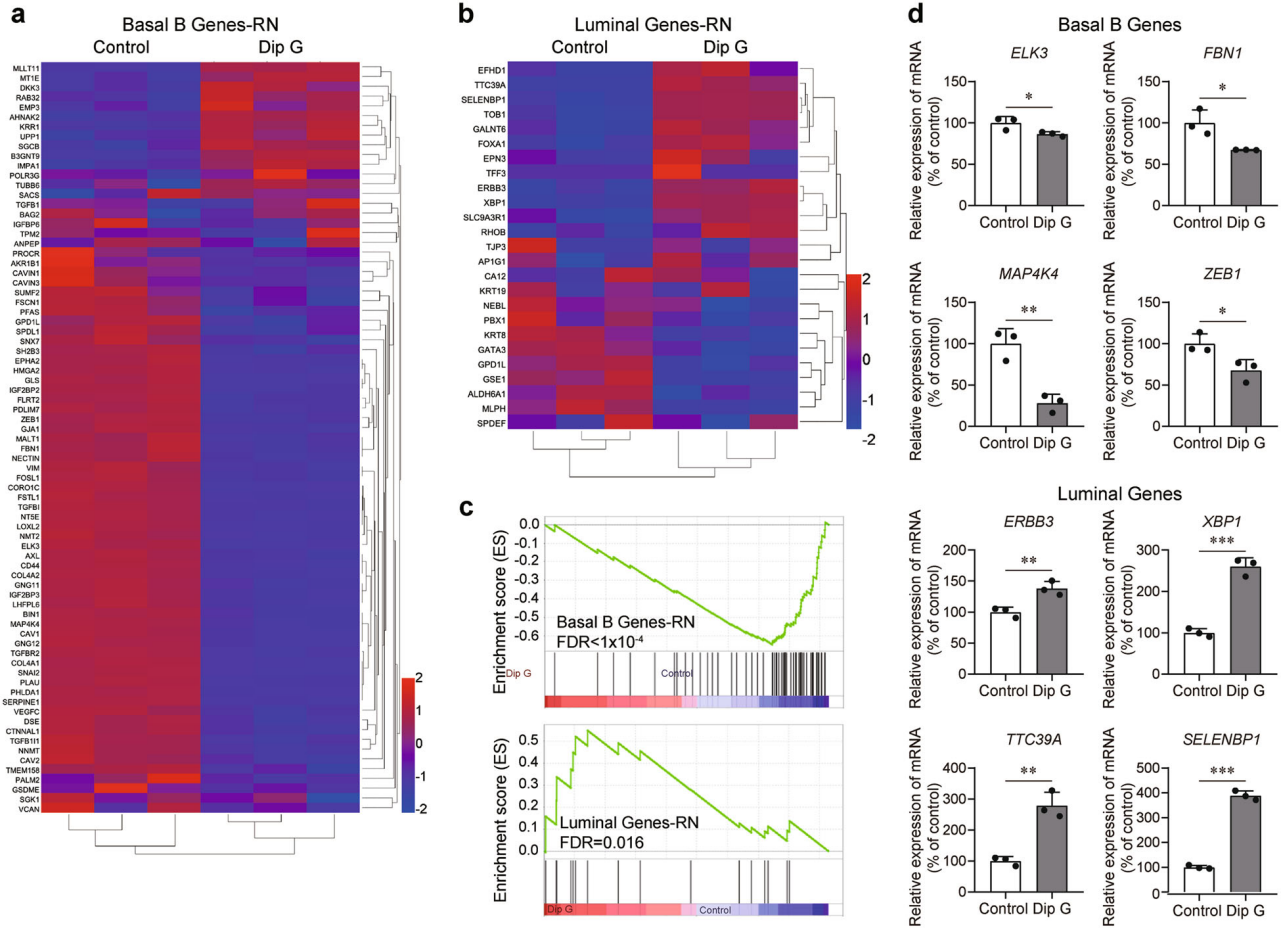
Induction of a partial transcriptome shift from basal to luminal gene signatures by Dip G in basal-like breast cancer cells. MDA-MB-231 cells were treated with or without Dip G (15 μM) for 24 h. mRNA expression was detected using RNA sequencing. **a**, **b** Heat maps depicting expression changes of the marker genes classified as basal B (**a**) and luminal (**b**) by Neve et al. (RN)^1^. A propensity of blue or red is indicative of a directional shift in the global expression of the marker gene. **c** GSEA enrichment plots utilizing a subset of the basal B and luminal differentiating gene sets determined by Neve et al. (RN)^1^. Vertical lines represent individual genes of the respective classifier that contribute to the enrichment scores. **d** Quantitation of gene expression changes for a panel of differentially expressed basal B and luminal marker genes performed using real-time RT-PCR. Data are shown as the mean ± S.D. of three independent experiments. *P < 0.05, **P < 0.01, ***P < 0.001.

Next, the gene expression profile of MDA-MB-231 (classified as basal B^1^) cells treated with Dip G was analyzed using HiSeq deep sequencing. After 24 h of treatment, Dip G significantly downregulated 61 of 77 (79.2%) of previously defined basal B cell line classifier genes, while concomitantly upregulating 9 of 25 (36%) of luminal classifier genes (Table 1)^1^. Ordered heat maps depicting expression changes of classifiers for basal B and luminal cells are shown in Fig. 2a, b, respectively. Based on a large subset of the classifier genes defined by Neve et al. (RN)^1^ that can differentiate between basal A, basal B and luminal subtypes, Gene Set Enrichment Analysis (GSEA) revealed a significant shift from the basal B to the luminal signature (Fig. 2c). Dip G treatment decreased the enrichment of basal B gene expression [false discovery rate (FDR) *q* < 1 × 10^4^], while concomitantly increasing the enrichment of luminal gene expression (FDR *q* = 0.016). As shown in Table 2, there was no enrichment of basal A genes. A combined gene set of basal A and B genes was also significantly enriched (FDR *q* < 1 × 10^4^). To confirm the HiSeq deep-sequencing data, expression changes in a subset of the significantly altered basal B and luminal marker genes were quantified using real-time RT-PCR. The decrease in basal B gene expression and increase in luminal gene expression upon Dip G treatment, was confirmed in MDA-MB-231 cells (Fig. 2d). Taken together, these results suggest that Dip G has the potential to trigger the switch from a basal-like to a luminal-like breast cancer subtype.

**Table 1:** Basal B and Luminal classifier genes whose expression is changed after Dip G treatment in MDA-MB-231 cells (*p* < 0.05)^a^.

Basal B (decreased)				Luminal (increased)
*HMGA2*	*TGFBR2*	*NNMT*	*FSCN1*	*EFHD1*
*SERPINE1*	*GJA1*	*MAP4K4*	*SNX7*	*TTC39A*
*PLAU*	*IGF2BP3*	*BIN1*	*GPD1L*	*FOXA1*
*SNAI2*	*FBN1*	*TGFB1I1*	*CAVIN3*	*ERBB3*
*LOXL2*	*FOSL1*	*CAV2*	*PFAS*	*SELENBP1*
*NT5E*	*LHFPL6*	*AXL*	*AKR1B1*	*XBP1*
*COL4A2*	*PDLIM7*	*FSTL1*	*SPDL1*	*TOB1*
*PHLDA1*	*TGFBI*	*CTNNAL1*	*CAVIN1*	*RHOB*
*COL4A1*	*GNG11*	*DSE*	*SGK1*	*GALNT6*
*VEGFC*	*FLRT2*	*EPHA2*	*GSDME*	
*IGF2BP2*	*NECTIN3*	*CORO1C*	*SUMF2*	**36% of total Lum**
*NMT2*	*GNG12*	*VIM*	*PROCR*	
*CD44*	*ZEB1*	*PALM2*	*IGFBP6*	
*CAV1*	*MALT1*	*TMEM158*	*TPM2*	
*GLS*	*ELK3*	*SH2B3*	*BAG2*	
			*TUBB6*	
	**79.2% of total BasB**			

^a^Classifier genes based on Neve et al.^1^.

**Table 2:** GSEA of classifier gene lists that are discriminatory luminal versus basal-like breast cancer molecular subtypes^a^.

Name	ES	NES	NOM *p*-value	FDR *q*-value	FWER *p*-value
Lum-RN	0.548	1.553	0.035	0.016	0.045
BasA-RN	0.234	0.983	0.477	0.482	0.48
BasB-RN	−0.649	−2.867	0	<1 × 10^−4^	0
BasAB-RN	−0.483	−2.393	0	<1 × 10^−4^	0

^a^Classifier genes based on Neve et al.^1^.

*ES* enrichment score, *NES* normalized enrichment score, *NOM* nominal, *FDR* false discovery rate, *FWER* familywise-error rate.

### GABARAPL1 is associated with Dip G-induced phenotype switch of breast cancer cells

**Fig. 3:**
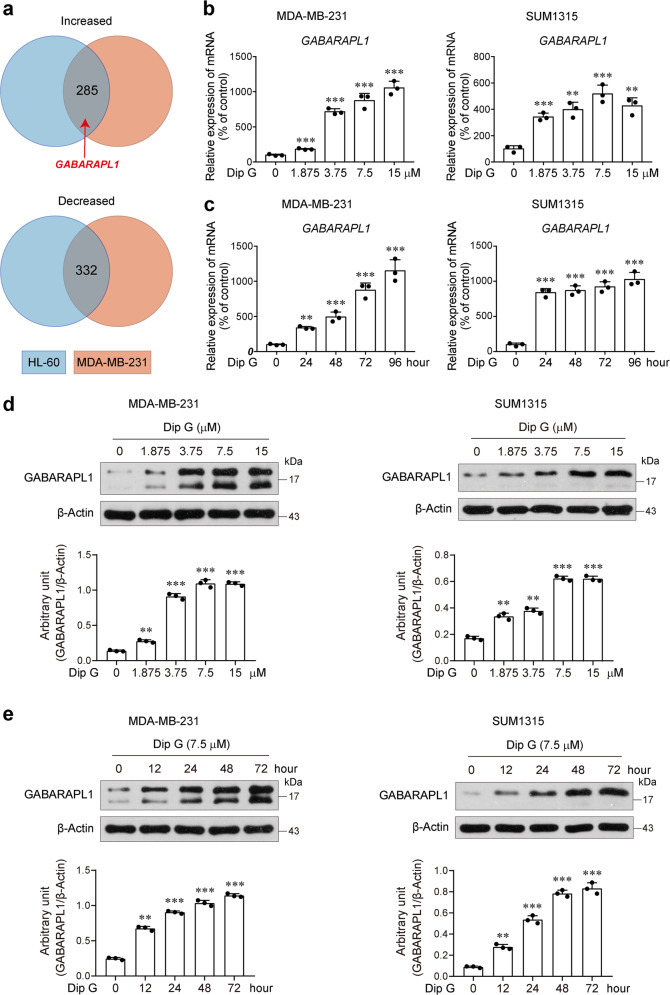
Upregulation of GABARAPL1 expression by Dip G in basal-like breast cancer cells. **a** Overlap of the common differentially regulated genes in Dip G-treated HL-60 and MDA-MB-231 cells. *GABARAPL1* is included in the common upregulated genes. **b**, **c** Cells were treated with various concentrations of Dip G for 72 h (**b**) or in the presence of Dip G (7.5 μM) for the indicated times (c). The mRNA levels of GABARAPL1 were determined by real-time RT-PCR. β-Actin was used as an internal control. **e**, **f** Cells were treated with various concentrations of Dip G for 24 h (**e**) or in the presence of Dip G (7.5 μM) for the indicated times (**f**). The protein levels of GABARAPL1 were determined by western blot. β-Actin was used as a loading control. Data are shown as the mean ± S.D. of three independent experiments. **P < 0.01, ***P < 0.001 versus the control group without Dip G treatment.

Our previous study reported that Dip G-induced myeloid differentiation of AML cells^22^. To investigate the unique molecular mechanism underlying Dip G-induced luminal differentiation of basal-like breast cancer cells, we compared gene expression changes in Dip G-treated MDA-MB-231 and the AML cell line HL60. Among the common differentially regulated genes, the most significantly regulated gene was found to be *GABARAPL1* (Fig. 3a and Supplementary Table S1). Dip G upregulated the mRNA expression of GABARAPL1 in a dose- and time-dependent manner in both MDA-MB-231 and SUM1315 cells (Fig. 3b, c). Western blotting results confirmed the GABARAPL1-inducing function of Dip G (Fig. 3d, e). In addition, analysis of the transcriptional profiles of 1097 breast tumors collected from The Cancer Genome Atlas (TCGA) revealed that the expression of *GABARAPL1* was substantially lower than that in normal breast tissues (Fig. S4), indicative of an essential role of GABARAPL1 downregulation during breast cancer progression.

**Fig. 4:**
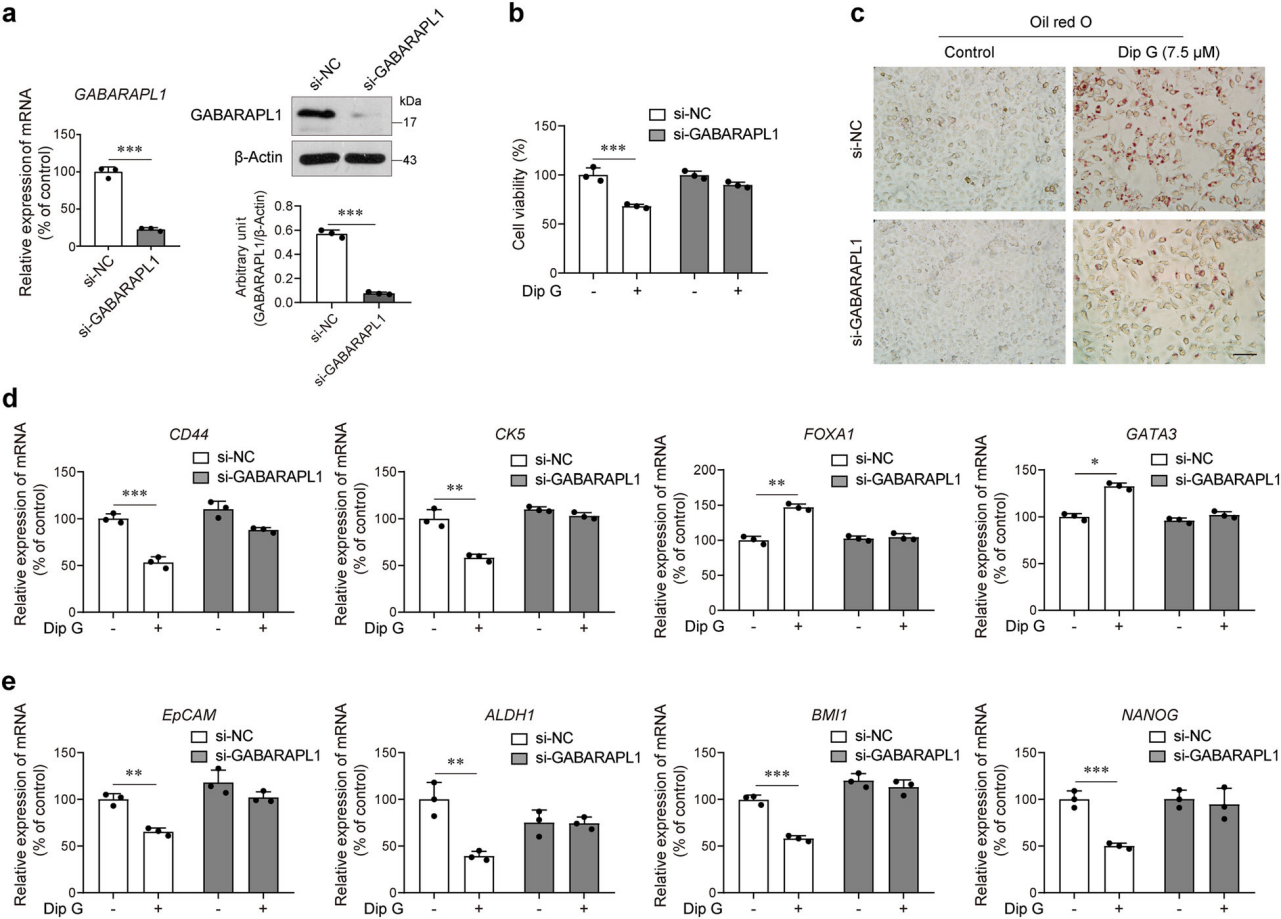
Knockdown of GABARAPL1 attenuates Dip G-induced cell differentiation. SUM1315 cells were transfected with NC-siRNA or siRNA targeting GABARAPL1 for 24 h and then treated with Dip G (7.5 μM) for 72 h. **a** The mRNA and protein levels of GABARAPL1 upon knockdown with siRNA. **b** Cell viability was tested by a trypan blue exclusion assay. **c** Oil Red O staining for neutral fat contents. Scale bar: 50 μm. **d** Expression of basal *CD44* and *CK5* and luminal *FOXA1* and *GATA3* genes. **e** Expression of stemness-associated genes *EpCAM*, *ALDH1*, *BMI1*, and *NANOG*. Data are shown as the mean ± S.D. of three independent experiments. *P < 0.05, **P < 0.01, ***P < 0.001.

Next, we set out to determine the functional implications of the induced GABARAPL1 expression in basal-like breast cancer cells. We silenced GABARAPL1 in SUM1315 cells and confirmed the decrease in both mRNA and protein levels of GABARAPL1 (Fig. 4a). Silencing GABARAPL1 strikingly reversed Dip G-induced inhibition of cell growth (Fig. 4b). Oil red O staining results showed that knockdown of GABARAPL1 attenuated the intensity of Oil red O after Dip G treatment (Fig. 4c). More importantly, Dip G failed to decrease the expression of the basal-like genes *CD44* and *CK5* and to increase the expression of the luminal-like genes *FOXA1* and *GATA3* in the cells with GABARAPL1 silenced (Fig. 4d). The reduction in the expression of stemness-associated genes by Dip G was also rescued by the knockdown of GABARAPL1 (Fig. 4e). Although autophagy is reported to be involved in cell differentiation and GABARAPL1 exerts an important role in the late stages of the formation of the autophagosome^15,21^, we did not observe an effect of Dip G on the autophagy level in base-like breast cells, as evidenced by the failure in the conversion of LC3B-I to II and the degradation of GFP-LC3 (Fig. S5). These results suggest that GABARAPL1 might be involved in the Dip G-induced phenotype switch of breast cancer in a novel manner independent of the enhancement of autophagy.

### Dip G-induced GABARAPL1 expression is dependent on the upregulation of ERβ

**Fig. 5:**
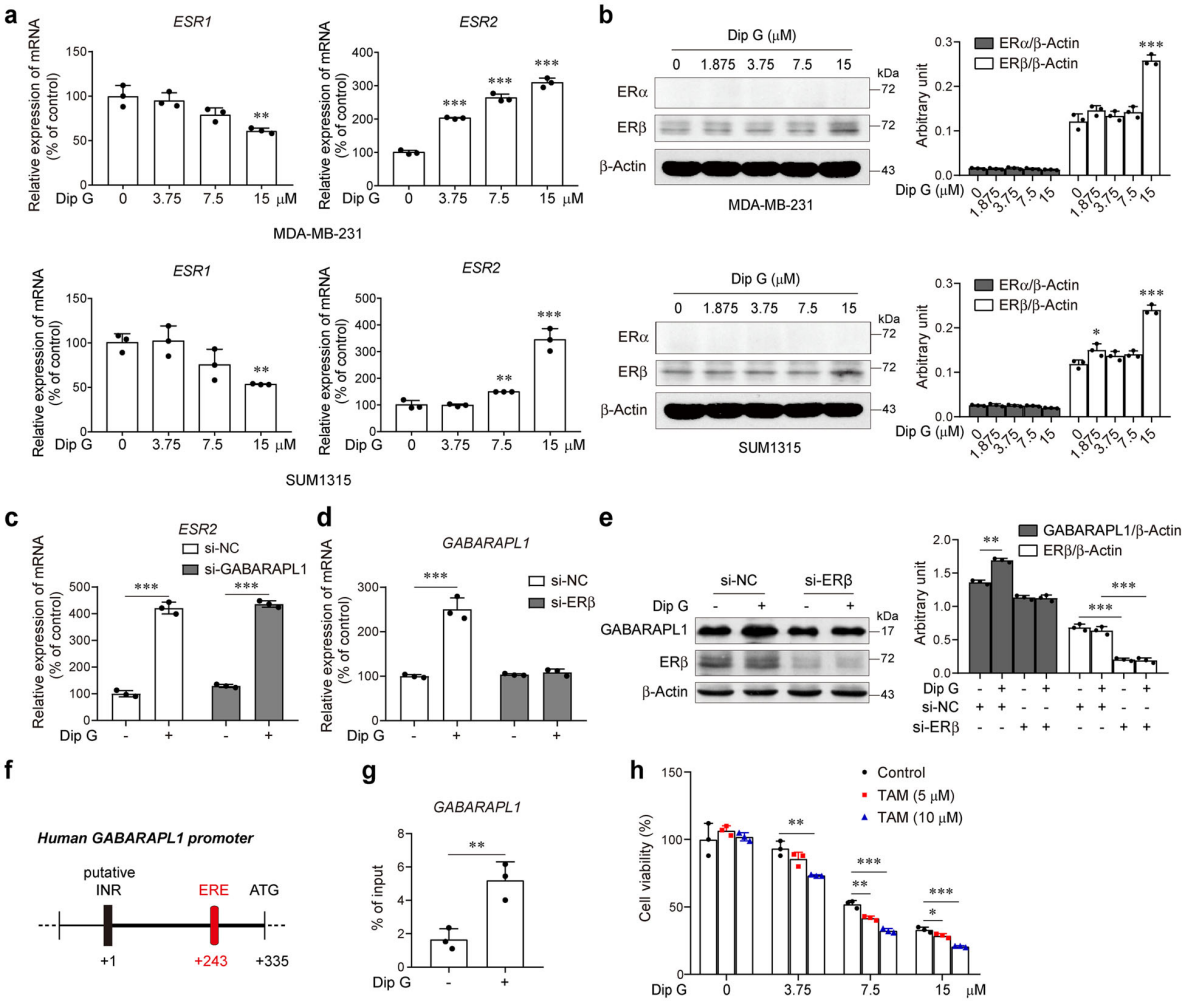
Upregulation of GABARAPL1 expression by Dip G is dependent on ERβ. **a** Cells were treated with various concentrations of Dip G for 72 h. The mRNA levels of *ESR1* and *ESR2* were determined by real-time RT-PCR. β-Actin was used as an internal control. **P < 0.01, ***P < 0.001 versus the control group without Dip G treatment. **b** Cells were treated with various concentrations of Dip G for 24 h. The protein levels of ERα and ERβ were determined by western blot. β-Actin was used as a loading control. **c** SUM1315 cells were transfected with NC-siRNA or siRNA targeting GABARAPL1 for 24 h and then treated with Dip G (7.5 μM) for 72 h. The mRNA levels of *ESR2* were determined. **d**, **e** SUM1315 cells were transfected with NC-siRNA or siRNA targeting ERβ for 24 h and then treated with Dip G (7.5 μM) for 24 h. The mRNA (**d**) and protein (**e**) levels of GABARAPL1 were determined. **f** Diagram of the location of ERE in the human GABARAPL1 gene promoter region. ERE: Estrogen Responsive Element; INR: transcription initiator element; ATG: translation initiation codon. **g** MDA-MB-231 cells were treated with Dip G (7.5 μM) for 24 h. ChIP assays were performed using an antibody against ERβ, followed by real-time RT-PCR with primers designed for the ERE in the GABARAPL1 promoter region. (**h**) MDA-MB-231 cells were pretreated with various concentrations of Dip G for 72 h and then treated with tamoxifen for another 48 h. Cell viability was determined by MTT assay. Data are shown as the mean ± S.D. of three independent experiments. **P < 0.01, ***P < 0.001.

It has been reported that Dip G targets the CHIP E3 ubiquitin ligase and reciprocally controls ERα and ERβ protein stability in breast cancer cells, and that GABARAPL1 is identified as an early estrogen-induced gene^15,24^. To further investigate whether the induction of GABARAPL1 by Dip G is linked to its control of ER, we tested the effects of Dip G on the expression of ERα and ERβ in MDA-MB-231 and SUM1315 cells. In line with a previous study^24^, Dip G enhanced the transcription of *ESR2*, which encodes ERβ, while concomitantly attenuating the transcription of *ESR1*, which encodes ERα (Fig. 5a). Western blotting analysis confirmed the increase in ERβ protein levels, although ERα was not detected (Fig. 5b). Silencing GABARAPL1 had no effect on Dip G-induced *ESR2* transcription (Fig. 5c). In contrast, the induction of *GABARAPL1* transcription by Dip G was completely reversed by knockdown of ERβ (Fig. 5d and Fig. S6). A similar result was obtained for GABARAPL1 protein expression (Fig. 5e), suggesting that ERβ could act upstream of GABARAPL1. Indeed, sequence analysis of the human *GABARAPL1* gene revealed the presence of ERE (estrogen responsive element) in the promoter regions (Fig. 5f). We performed a ChIP assay with an anti-ERβ antibody and real-time RT-PCR analysis in MDA-MB-231 cells. Dip G dramatically enhanced the recruitment of ERβ to the *GABARAPL1* promoter regions (Fig. 5g), supporting the direct regulation of *GABARAPL1* transcription. Furthermore, we found that pretreatment with Dip G significantly increased the sensitivity of MDA-MB-231 cells to tamoxifen-induced growth arrest (Fig. 5h). These results suggest that Dip G stimulated the expression of GABARAPL1 in an ERβ-dependent manner, leading to a luminal-like phenotype denoted by an increased sensitivity to tamoxifen.

### Dip G suppresses the in vivo growth of basal-like breast cancer cells and prompts sensitization to tamoxifen therapy

**Fig. 6:**
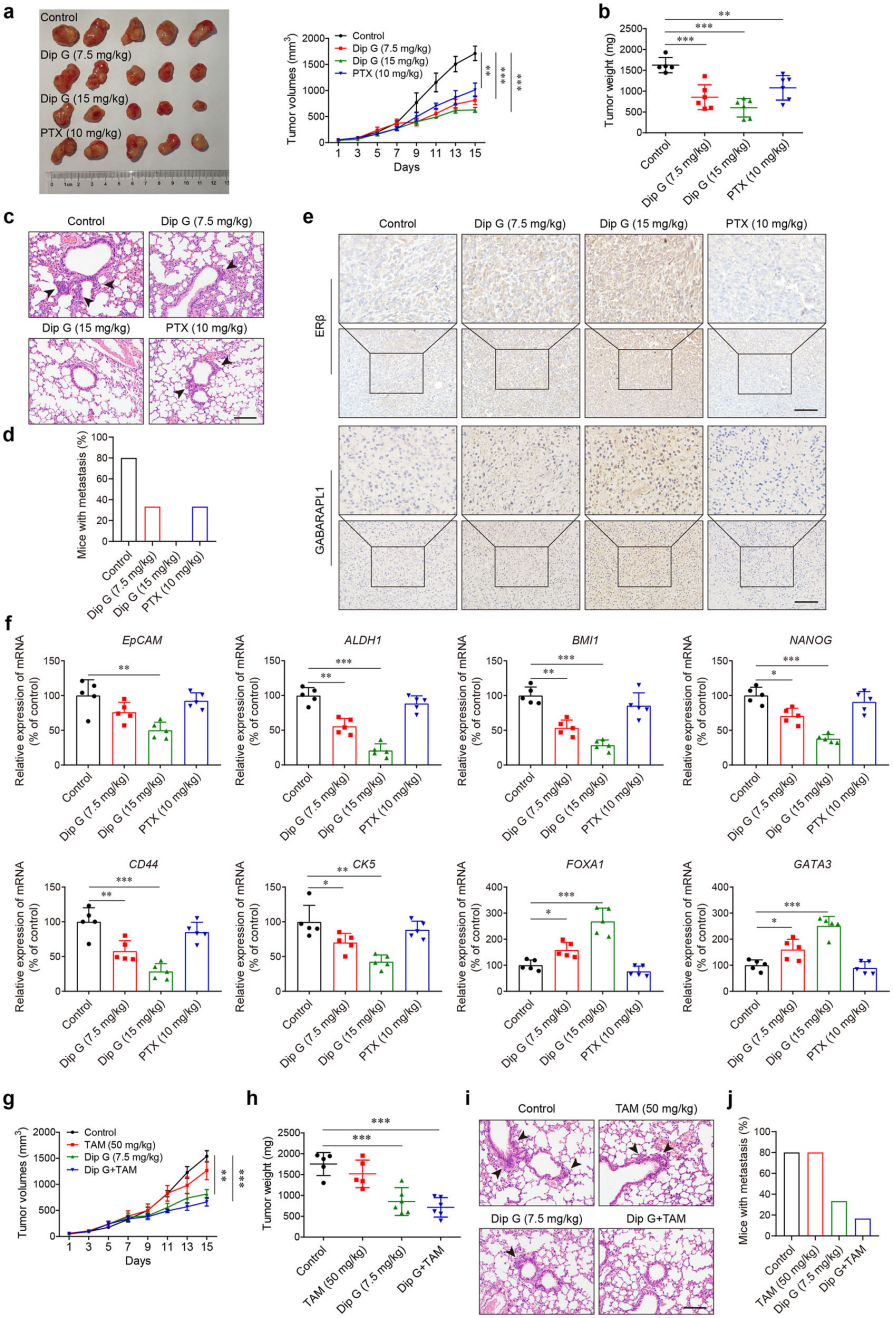
In vivo therapeutic efficacy of Dip G by triggering basal-like breast tumor differentiation. MDA-MB-231 cells (2 × 10^6^) were inoculated into the mammary fat pads of female nude mice. After 10 days, mice (*n* = 6) were treated with vehicle control (olive oil), Dip G (7.5 or 15 mg/kg/day) or PTX (10 mg/kg/7 days) intraperitoneally for an additional 14 days. **a** Tumor growth curves. Left panel: representative images of the tumors at the end of the experiments. **b** Tumor weight on day 15. **c** Representative images of lung sections stained with H&E. The lung tissues were excised on day 15. Black arrow, metastatic foci in the lungs. Scale bar, 100 μm. **d** The proportion of mice with lung metastasis in each group. **e** Tumor sections were stained with antibodies against ERβ and GABARAPL1. Upper, enlarged images of rectangles. Scale bar, 100 μm. **f** The mRNA levels of the indicated genes in tumors excised on day 15. β-Actin was used as an internal control. **g**–**j** The mice carrying orthotopically transplanted MDA-MB-231 tumors were treated with Dip G (7.5 mg/kg/day, intraperitoneally), tamoxifen (50 mg/kg/day, orally) or a combination of the two. **g** Tumor growth curves. **h** Tumor weight. **i** Representative images of lung sections stained with H&E. Scale bar, 100 μm. **j** The proportion of mice with lung metastasis in each group. *P < 0.05, **P < 0.01, ***P < 0.001.

To evaluate the therapeutic efficacy of Dip G in basal-like breast tumors, we inoculated MDA-MB-231 cells into the mammary fat pads of female athymic nude mice, which were treated 10 days later with intraperitoneal injection of vehicle control, Dip G (7.5 or 15 mg/kg/day) or paclitaxel (PTX, 10 mg/kg/7 days) over a period of 2 weeks. As shown in Fig. 6a, Dip G treatment inhibited tumor growth in a dose-dependent manner. Dip G (7.5 mg/kg) showed an inhibitory effect on the development of tumors to a higher extent than PTX (10 mg/kg). At day 15 after administration, the average tumor weight was approximately two- or three-fold less in the mice treated with 7.5 or 15 mg/kg of Dip G compared with the vehicle controls (Fig. 6b). H&E staining showed extensive pulmonary metastasis in vehicle control mice (Fig. 6c), and ~80% of the control mice developed lung metastases (Fig. 6d). In contrast, Dip G treatment markedly reduced the incidence of metastasis, and no metastatic foci were found in the lungs of mice treated with 15 mg/kg Dip G. Furthermore, positive immunostaining for ERβ and GABARAPL1 revealed that the Dip G-treated tumors had a dose-dependent increase in these proteins (Fig. 6e). Dip G treatment also reduced the mRNA expression of both stemness-associated genes and basal-like genes *CD44* and *CK5*, while increasing the expression of the luminal-like genes *FOXA1* and *GATA3* in the tumors (Fig. 6f). However, the PTX-treated tumors showed no change compared with the controls.

To demonstrate the utility of Dip G for sensitization of basal-like cancer tumors to the action of tamoxifen, we assessed the therapeutic potential of the combination of Dip G and tamoxifen in an MDA-MB-231 orthotopic-transplant model. As expected, treatment with tamoxifen alone was unable to influence the in vivo growth of MDA-MB-231 tumors (Fig. 6g). In contrast, combined administration of tamoxifen with 7.5 mg/kg of Dip G led to significant growth inhibition and tumor weight reduction (Fig. 6g, h). In addition, the frequency of pulmonary metastasis was greatly decreased following tamoxifen therapy in combination with Dip G (Fig. 6i, j), suggesting that the induction of ERβ and GABARAPL1 indeed invoked sensitivity to the action of tamoxifen.

## Discussion

Due to the lack of therapeutic targets, breast cancer of the basal-like, hormone receptor-negative subtype remains an unmet clinical challenge. Thus, new treatment approaches are urgently needed. In this study, we demonstrate that the natural product Dip G exhibits a robust growth inhibitory activity in basal-like breast cancer cell lines by inducing cell differentiation both in vitro and in vivo. More importantly, Dip G treatment resulted in the conversion of basal-like breast cancer cells into a luminal state that rendered sensitivity to tamoxifen therapy. Rather than searching for new drugs targeting genetic or epigenetic changes, pharmacological strategies that induce a switch from basal-like breast cancer to a luminal subtype may be a promising option based on the high degree of heterogeneity and plasticity in basal-like breast cancer cells. Such differentiation therapy is advantageous over traditional chemotherapy which indiscriminately killing proliferating cells. Abundant studies are currently in progress trying to use differentiation agents such as ATRA, miR-100 and histone deacetylase inhibitors in breast cancer^26–28^. miR-100 promotes breast cancer cell differentiation by changing a basal-like phenotype into a luminal phenotype and boosts the basal-like breast cancer response to hormonal therapy through the expression of ER^27^. In addition, blockade of PDGF-CC through pharmacological means has been reported to prompt sensitization of previously resistant basal-like breast tumors to the action of endocrine therapy by inducing ER^10^. To our knowledge, Dip G is the first natural product that triggers the phenotype switch in basal-like breast cancer. Given its feasibility of totally synthesis, Dip G may be developed as a novel differentiation agent for patients with basal-like breast cancer.

A phenotype switch from basal-like to luminal breast cancer can be achieved through the expression of FOXA1, GATA3 or ESR1^11–13^. FOXA1 is essential for the transcriptional activation of luminal genes and repression of basal genes in breast cancers^13,29,30^. GATA3, another luminal subtype-specific transcription factor, has been proposed to play a similar role as FOXA1^31,32^. Indeed, Dip G increased the expression of *FOXA1* and *GATA3*, while suppressing the expression of the basal-like genes *CD44* and *CK5* both in vitro and in vivo. The genome-wide expression analysis showed that *FOXA1* expression was 2.55-fold higher in MDA-MB-231 cells treated with Dip G than in the untreated control (See GSE148100). Although Dip G induced the expression of a subset of, but not all of luminal genes, GSEA revealed an induction of a molecular signature with a more luminal pattern upon Dip G treatment. Decreased aggressiveness of basal-like breast cancer cells, as evidenced by a growth arrest and reduction in migration and stemness, further suggests that Dip G is capable of inducing less differentiated breast cancer cells to acquire phenotypic characteristics of more differentiated or luminal cells.

In this study, our findings reveal a previously unappreciated role for GABARAPL1 as a determinant of the molecular subtype of breast cancer. Dip G has a robust GABARAPL1-inducing activity in basal-like breast cancer cells. Transient GABARAPL1 silencing caused the failure of Dip G to increase the expression of *FOXA1* and *GATA3*, accompanied by the rescued cell viability and stemness. Interestingly, upregulation of GABARAPL1 has also been observed during ATRA-induced neutrophil differentiation in AML^21^ and Dip G-induced AML myeloid differentiation (unpublished data), indicative of the link between GABARAPL1 and cell differentiation. It is well known that GABARAPL1 associates with autophagic vesicles and regulates autophagic flux^33^. However, we did not observe the elevated autophagy levels in base-like breast cells treated with Dip G, suggesting that GABARAPL1-mediated autophagy could not account for its function in cell differentiation. Similarly, Poillet-Perez et al.^34^ reported that GABARAPL1 tumor suppressive function is independent of its conjugation to autophagosomes in breast cancer cells. Rather than having autophagy-regulating action, GABARAPL1 could play a differentiation-inducing role in other mechanisms that remain to be investigated.

Although ESR1 is a luminal lineage-driving transcription factor, Dip G attenuated its transcription and showed no detectable effect on ERα protein level. In contrast, Dip G enhanced the expression of ESR2 at both the mRNA and protein levels, suggesting that Dip G acts as a dual-functional moiety as described previously^24^. In fact, a previous study reported that instead of targeting ER, Dip G targets the CHIP E3 ubiquitin ligase shares by ERα and ERβ, and increases ERβ protein stability, while decreasing ERα protein levels in breast cancer cells^24^. Both ERα and ERβ mediate estrogen signaling by binding to the ERE of target genes^35^. ChIP assays showed that Dip G remarkably enhanced the recruitment of ERβ to the *GABARAPL1* promoter regions. Dip G-induced GABARAPL1 expression was reversed by knocking down ERβ, while silencing GABARAPL1 showed no effect on the upregulation of ERβ by Dip G. It is thus likely that Dip G induces basal-like breast cancer cell differentiation via an ERβ-dependent induction of GABARAPL1. Many studies have reported the link between high ERβ expression in clinical samples of breast cancer and better prognosis^36^. Studies in cell lines and a preclinical breast cancer animal model also suggest a beneficial effect of ERβ^37–39^. Moreover, ERβ expression may correlate with a favorable response to endocrine therapy^40,41^. There is an ongoing clinical trial to investigate the effectiveness of adjuvant endocrine therapy for operable ERβ positive, ERα/PR negative, HER-2 negative breast cancer (https://clinicaltrials.gov/ct2/show/NCT02089854). Indeed, we found that Dip G was useful as a combination partner to tamoxifen therapy in MDA-MB-231 tumors.

In summary, our study demonstrated that the natural small-molecule Dip G induces cell differentiation by converting basal-like breast cancer cells into a luminal state that renders sensitivity to tamoxifen therapy. In addition, our findings identified GABARAPL1 as an ERβ-dependent regulator of the molecular subtype of breast cancer. This work reveals new therapeutic opportunities for refractory basal-like breast cancer.

## Materials and methods

### Reagents and antibodies

Dip G (>98% purity) was isolated as previously described^22^. Chloroquine, oil red O, sodium butyrate (NaB), methyl thiazolyl tetrazolium (MTT) and tamoxifen (TAM) were purchased from Sigma-Aldrich (St. Louis, MO). Z-VAD-FMK and necrostatin-2 were from Selleck (Houston, TX). Anti-E-cadherin (14472), anti-vimentin (5741), anti-N-cadherin(13116), anti-p-β-catenin (Ser33/37/Thr41) (9561), anti-p-β-catenin (Thr41/Ser45) (9565), anti-p-β-catenin (Ser552) (5651), anti-p-β-catenin (Ser675) (4176), anti-β-catenin (8480), anti-c-Myc (18583), anti-LC3B (3868) and anti-GABARAPL1 (26632) were purchased from Cell Signaling Technology (Beverly, MA). Polyclonal anti-GABARAPL1 (11010-1-AP) was from Proteintech Group (Chicago, IL). Anti-ERα (sc-8002), anti-ERβ (sc-373853 X) and anti-β-Actin (sc-47778) were from Santa Cruz Biotechnology (Santa Cruz, CA). Anti-SQSTM1/p62 (ab101266) was from Abcam (Cambridge, UK). CD24 monoclonal antibody (FITC) (11-0247-42), CD44 monoclonal antibody (PE-eFluor 610) (61-0441-80), Lipofectamine 3000 and Lipofectamine RNAi MAX were purchased from Thermo Fisher Scientific (Waltham, MA).

### Cell culture and transfection

Human basal-like breast cancer cell lines MDA-MB-231, SUM1315 and MDA-MB-468 were purchased from the Cell Bank Type Culture Collection of the Chinese Academy of Sciences (Shanghai, China), who provided an authentication certificate. All of the cells were cultured in DMEM medium (Life Technologies, Grand Island, NY) containing 10% FBS (GIBCO, Grand Island, NY), 100 mg/ml streptomycin and 100 U/ml penicillin at 37 °C in a humidified atmosphere with 5% CO_2_.

mCherry-GFP-LC3 plasmid was purchased from Addgene (Cambridge, MA). siRNA targeting GABARAPL1 and ERβ were from Santa Cruz Biotechnology. SUM1315 cells were transfected with mCherry-GFP-LC3 plasmid using Lipofectamine 3000. For RNA interference, SUM1315 cells were transfection with siRNA using Lipofectamine RNAi MAX according to the manufacturer’s instruction.

### Mice

6–8 weeks-old female BALB/c nude mice were obtained from the Model Animal Research Center of Nanjing University (Nanjing, China). All animal studies were carried out in compliance with the guidelines (Ministry of Science and Technology of China, 2006) and relevant ethical regulations of Nanjing University. All efforts were made to minimize the suffering of animals and the number of animals used.

### Real-time RT-PCR

RNA samples isolated from cell lines or tumors were reverse transcribed to cDNA and subjected to quantitative PCR, which was performed using the iQSYBR Green Supermix (Bio-Rad, Hercules, CA) and BioRad CFX96 Touch Real-Time PCR Detection System (Bio-Rad). The threshold cycle numbers were determined using the BioRad CFX Manager software V.5.0. The condition for amplification was 1 cycle at 95 °C for 2 min followed by 40 cycles at 95 °C for 15 s, 60 °C for 30 s, and 95 °C for 10 s. The primer sequences used in this study are listed in the Supplementary Table S2.

### Western blot

Western blot analysis was performed as previously described^22^.

### Oil red O staining

Cultured cells were fixed with 4% paraformaldehyde for 30 min, washed with PBS and soaked with 60% isopropanol for 15 s. Then, the cells were stained with 0.3% Oil-Red-O in 60% isopropanol for 30 min and washed with PBS and 60% isopropanol. Merge images were obtained using a Nikon inverted microscope (Tokyo, Japan).

### Wound-healing assay

In total, 5 × 10^5^ cells were seeded into six-well plates and incubated in the presence or absence of Dip G until 100% confluence was reached. The layer of cells was scratched with the tip of micropipette and washed with PBS to remove cellular debris. Images were photographed at 0 and 6 h using a Nikon inverted microscope (Tokyo). The distance of cell migration was analyzed using Image J software (NIH, Bethesda, MD).

### RNA sequencing

MDA-MB-231 cells were treated without or with 15 μM of Dip G for 24 h. The total RNA was extracted using the mirVana miRNA Isolation Kit (Thermo Fisher Scientific). RNA integrity was evaluated using the Agilent 2100 Bioanalyzer (Agilent Technologies, Santa Clara, CA). The samples with RNA Integrity Number ≥ 7 were subjected to the subsequent analysis. The libraries were constructed using TruSeq Stranded mRNA LTSample Prep Kit (Illumina, San Diego, CA) according to the manufacturer’s instruction. Then these libraries were sequenced on the Illumina sequencing platform (HiSeqTM 2500 or Illumina HiSeq X Ten). The transcriptome sequencing and analysis were conducted by OE biotech Co., Ltd. (Shanghai, China). The data were deposited at the Gene Expression Omnibus at NCBI under accession number GSE148100.

### Chromatin immunoprecipitation (ChIP)

ChIP assays were performed using Pierce Magnetic ChIP Kit (Thermo Fisher Scientific) according to the manufacturer’ s instruction. An antibody against ERβ was used for immunoprecipitation. The enrichment of ERβ at the promoter region of *GABARAPL1* was analyzed by real-time RT-PCR using the appropriated promoter primers. Relative quantification of target was normalized to input control. The primer sequences used in ChIP-PCR were as follows: GABARAPL1 forward, 5′-CGGACGTTTCTGCAGCTATTC-3′; GABARAPL1 reverse, 5′-GCAGGGCTTCCGAGATCC-3′.

### In vivo experiments

MDA-MB-231 cells (2 × 10^6^ cells in 20-μL PBS) were subcutaneously injected near the fat pad of the fourth mammary gland in the lower abdomen of nude mice. After 10 days, the mice bearing tumors (an average size of 50 mm^3^) were randomly divided into groups (*n* = 6 mice per group). Vehicle control (olive oil), Dip G (7.5 or 15 mg/kg/day) or paclitaxel (PTX, 10 mg/kg/7 days) were administered for 14 days by intraperitoneal injection. Tamoxifen (50 mg/kg/day) was orally administered for 14 days. Tumor volumes were measured every two days and calculated using the formula: *V* = 0.5236 × L1 × (L2)^2^, where L1 is the longer and L2 is the shorter tumor axis. At day 15, the mice were sacrificed and the tumor and lung tissues were excised.

### Histologic analysis and Immunohistochemistry

For histologic analysis, lung sections were stained with hematoxylin and eosin and photographed using a light microscope (Olympus). Immunostaining of GABARAPL1 and ERβ of tumor sections was performed using a Real Envision Detection kit (GeneTech, Shanghai) according to the manufacturer’ s instruction. Data analysis was performed blindly.

### Statistical analysis

Data are expressed as the means ± S.D. The Student’s *t* test was used to evaluate the difference between groups. In some experiments, statistical analyses were performed using one-way analysis of variance (ANOVA) followed by a post-hoc test. All the data were generated from at least three independent experiments. The investigators were not blinded to allocation during experiments and outcome assessment. There were no samples or animals excluded from the analysis. All statistical analyses were performed using the SPSS version 10.0 statistical software (SPSS, Chicago, IL). *P* < 0.05 was considered significant.

## Supplementary information

Supplementary Figure legends

Figure S1

Figure S2

Figure S3

Figure S4

Figure S5

Figure S6

Figure S7

Supplementary Table legends

Table S1

Table S2
