# Supplementary material for: Triggering a switch from basal- to luminal-like breast cancer subtype by the small-molecule diptoindonesin G via induction of GABARAPL1
Source: Cell Death Dis. 2020 Aug 15;11(8):635. doi: 10.1038/s41419-020-02878-z (PMC7429843; doi:10.1038/s41419-020-02878-z)
Supplement: Supplementary file 1 — Supplementary Figure legends [file 41419_2020_2878_MOESM1_ESM.docx]

**Triggering a switch from basal- to luminal-like breast cancer subtype by the small-molecule diptoindonesin G via induction of GABARAPL1**

Minmin Fan^1, *^, Jingwei Chen^1,*^, Jian Gao^1^, Wenwen Xue^1^, Yixuan Wang^1^, Wuhao Li^1^, Lin Zhou^1^, Xin Li^1^, Chengfei Jiang^2^, Yang Sun^1^, Xuefeng Wu^1^, Xudong Wu^1^, Huiming Ge^1^, Yan Shen^1^, Qiang Xu^1  ^

**Supplementary methods**

***Cell viability and proliferation assays***

Cells (1 x 10^5^ cells per well) were plated on a 12-well plate and treated with various concentrations of Dip G for indicated time courses. The number of cells were assessed in Trypan Blue dye exclusion method with manual cell counting using a hemocytometer (XB.K.25, QIUJING, Shanghai). For soft ager colony-formation assay, cells were pretreated with Dip G for 24 h. Then 1000 viable cells were seeded into 6-well plates and cultured for another 10 days. Cells were fixed with 4% paraformaldehyde and stained with crystal violet. The plates were photographed using a digital camera (Olympus). The number of colonies were detected at 40 × magnification using a Nikon inverted microscope. For EdU incorporation analysis, cells were treated with various concentrations of Dip G for indicated time courses. Then cells were collected and stain with EdU apollo 567 in vitro kit (Solarbio Life Sciences, Beijing, China) according to the manufacturer’s instruction.

***Flow cytometry analysis***

Cells were labeled with CD24-FITC and CD44-PE-eFluor 610 antibodies. The expression of CD24 and CD44 were analyzed using a FACSCalibur flow cytometer (Becton Dickinson, San Jose, CA). The data were processed by FlowJo software.

**Supplementary Figure Legends**

**Figure S1. Anti-proliferative effects of Dip G on basal-like breast cancer cells.** (a) MDA-MB-231, SUM1315 and MDA-MB-468 cells were treated with various concentrations of Dip G for 96 h, and the number of cells were counted using a Trypan Blue exclusion assay. (b) MDA-MB-231 and SUM1315 cells were pretreated with Dip G (7.5 μM) for 24 h. Then 1000 viable cells were cloned in soft agar and cultures for another 10 days. The six-well plates were photographed using a digital camera and the number of clones were counted. (c, d) SUM1315 cells were treated with various concentrations of Dip G for 72 h (c) or with Dip G (7.5 μM) for different time courses (d), the effects of Dip G on cell proliferation were using EdU staining assay. Scale bar, 50 μm. The data are the mean ± S.D. of three independent experiments. **P*<0.05, ***P*<0.01, ****P*<0.001 versus the control group without Dip G treatment.

**Figure S2. Dip G-mediated inhibition of cell proliferation is not attributable to apoptosis, necroptosis or autophagic cell death.** MDA-MB-231 cells were pretreated with various inhibitors (20 μM z-VAD-FMK, 30 μM Necrostatin-2, or 20 μM Chloroquine) for 2 h, then treated with Dip G (7.5 μM) for 72 h. Cell viability was determined by MTT assay. Data are shown as the mean ± S.D. of three independent experiments. ***P*<0.01.

**Figure S3.** **Dip G attenuates the stemness of basal-like breast cancer cells.** (a) MDA-MB-231 and SUM1315 cells were incubated with various concentrations of Dip G for 72 h or with Dip G (7.5 μM) for different time courses. CD24 and CD44 expression were detected using flow cytometry. (b) Cells were treated with various concentrations of Dip G for 24 h. The levels of indicated protein associated with the canonical Wnt/β-catenin signaling pathway were determined by western blot. β-Actin was used as a loading control. Data shown is representative of three experiments.

**Figure S4. GABARAPL1 is downregulated in breast cancer.** The mRNA expression of GABARAPL1 in normal tissues (n=114) and tumor tissues (n=1097) based on an analysis of the TCGA dataset. ****P*<0.001

**Figure S5. Dip G had no impact on the autophagy level in base-like breast cells.** (a) MDA-MB-231 and SUM1315 cells were treated with various concentrations of Dip G for 24 h. The protein levels of LC3B, p62 and GABARAPL1 were determined by western blot. β-Actin was used as a loading control. (b) SUM1315 cells were transfected with the mCherry-GFP-LC3 plasmid for 24 h and then treated with Dip G for 24 h or treated with EBSS for 4 h. The localization of mCherry-GFP-LC3 was detected by confocal immunofluorescence microscopy. Scan bar, 10 μm. Data shown is representative of three experiments.

**Figure S6. ESR2 mRNA level was knocked down by using siRNA.** SUM1315 cells were transfected with NC-siRNA or siRNA targeting ERβ for 48 h. The mRNA levels of ESR2 was determined by real-time RT-PCR. β-Actin was used as an internal control. Data are shown as the mean ± S.D. of three independent experiments. ***P*<0.01.

**Figure S7. The raw data of western blot for GABARAPL1.**
