# Supplementary figures and images for: Triggering a switch from basal- to luminal-like breast cancer subtype by the small-molecule diptoindonesin G via induction of GABARAPL1
Source: Cell Death Dis. 2020 Aug 15;11(8):635. doi: 10.1038/s41419-020-02878-z (PMC7429843; doi:10.1038/s41419-020-02878-z)

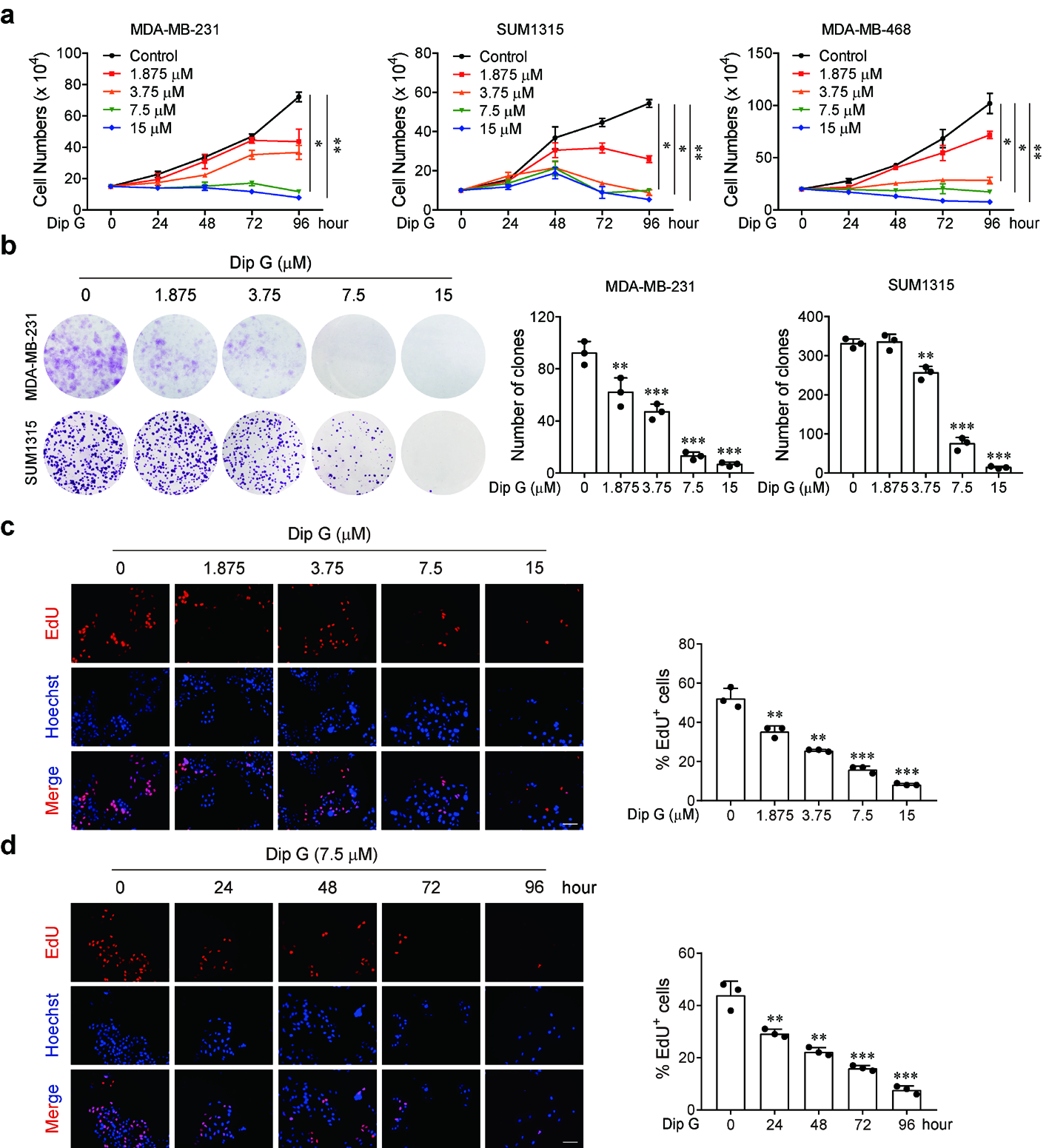

Supplement: Supplementary file 2 — Figure S1 [file 41419_2020_2878_MOESM2_ESM.tif]

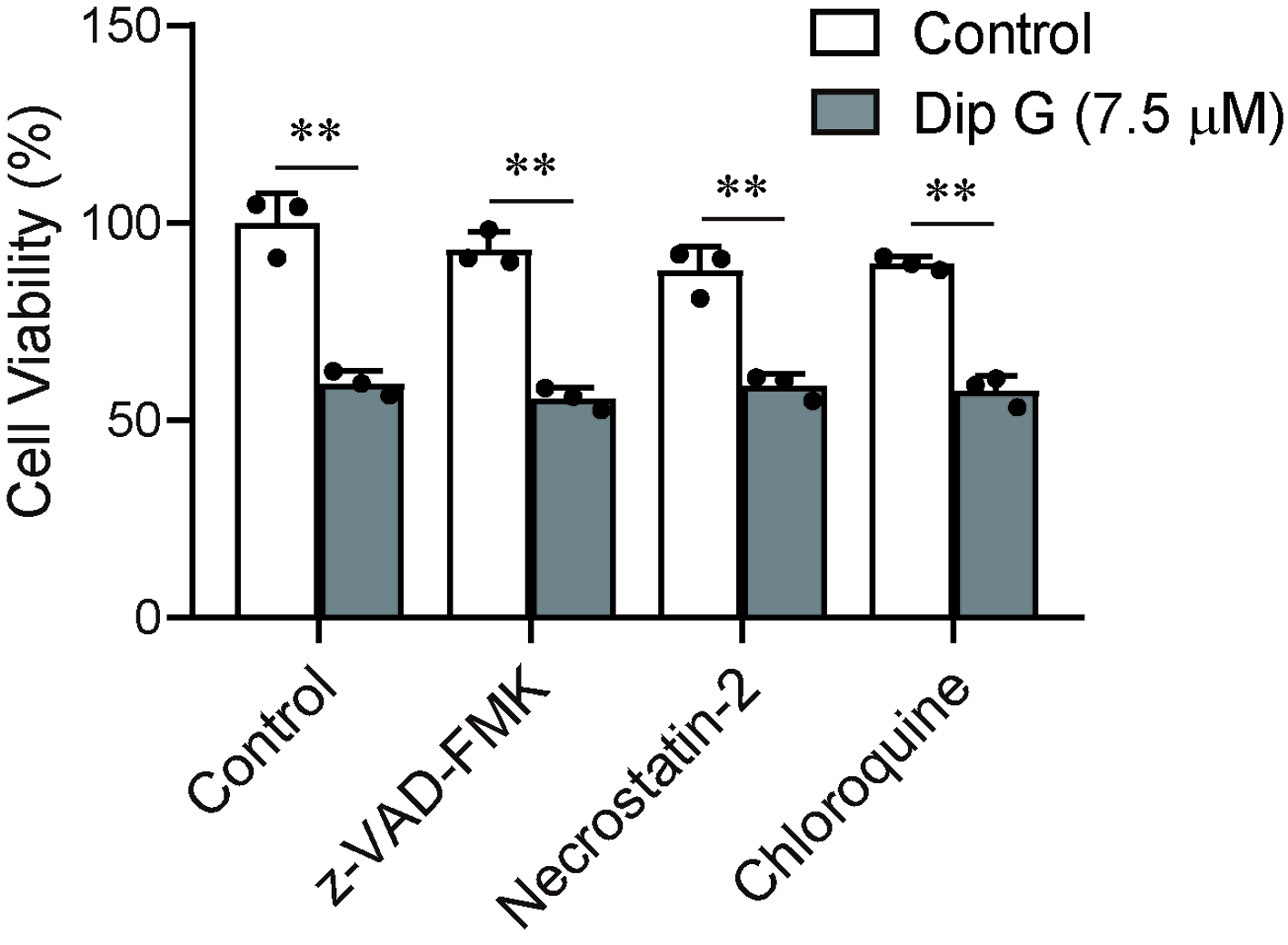

Supplement: Supplementary file 3 — Figure S2 [file 41419_2020_2878_MOESM3_ESM.tif]

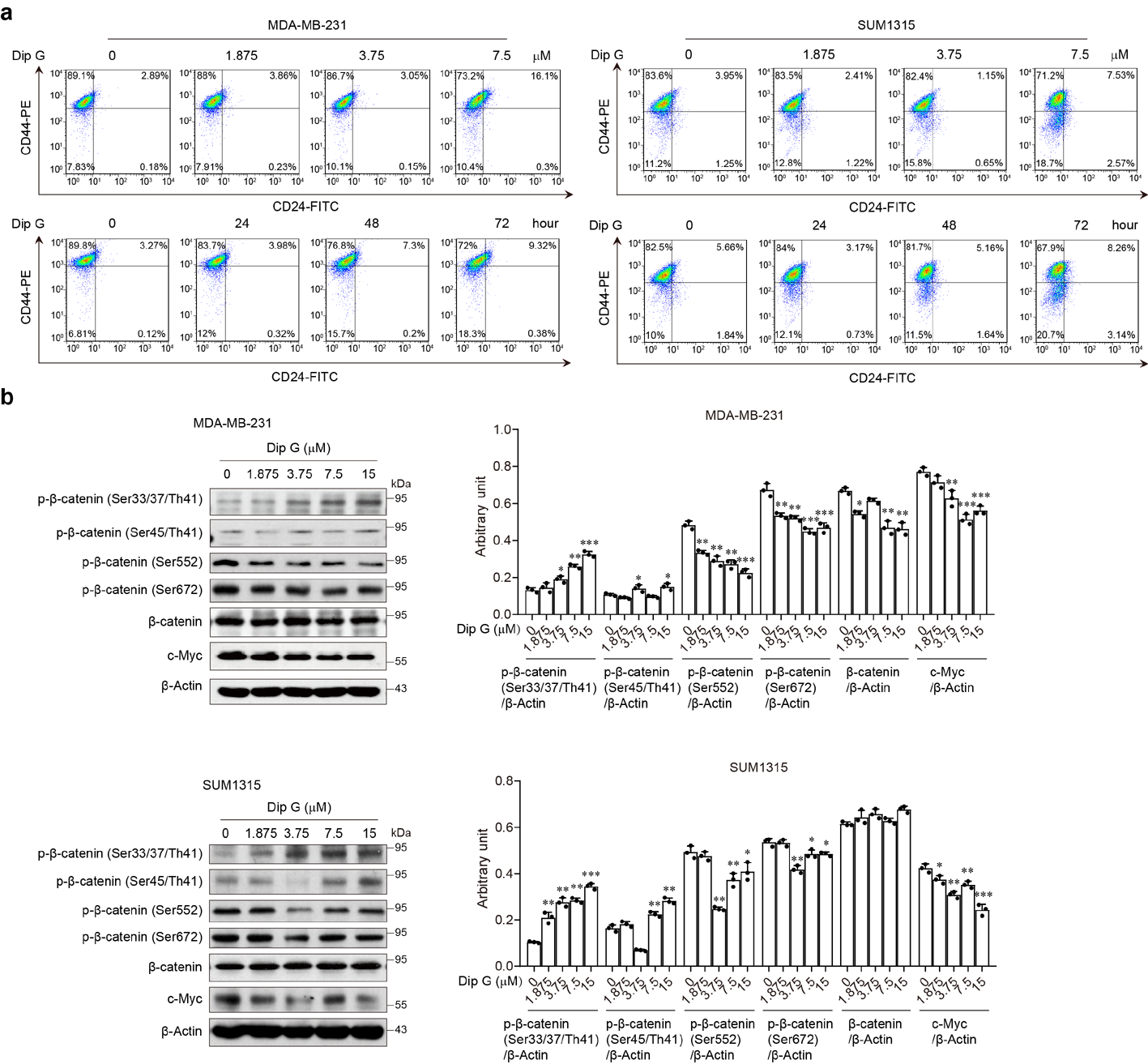

Supplement: Supplementary file 4 — Figure S3 [file 41419_2020_2878_MOESM4_ESM.tif]

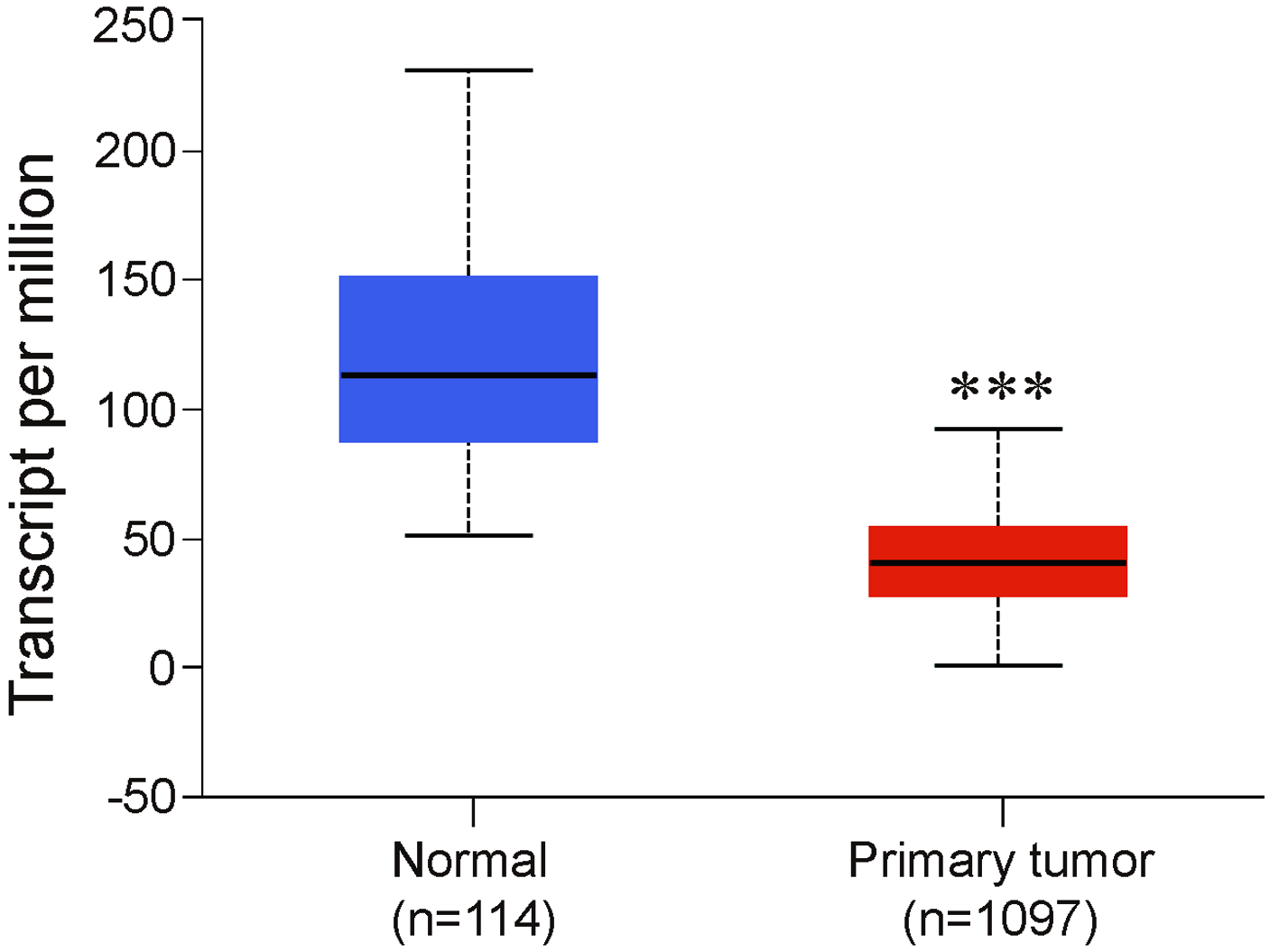

Supplement: Supplementary file 5 — Figure S4 [file 41419_2020_2878_MOESM5_ESM.tif]

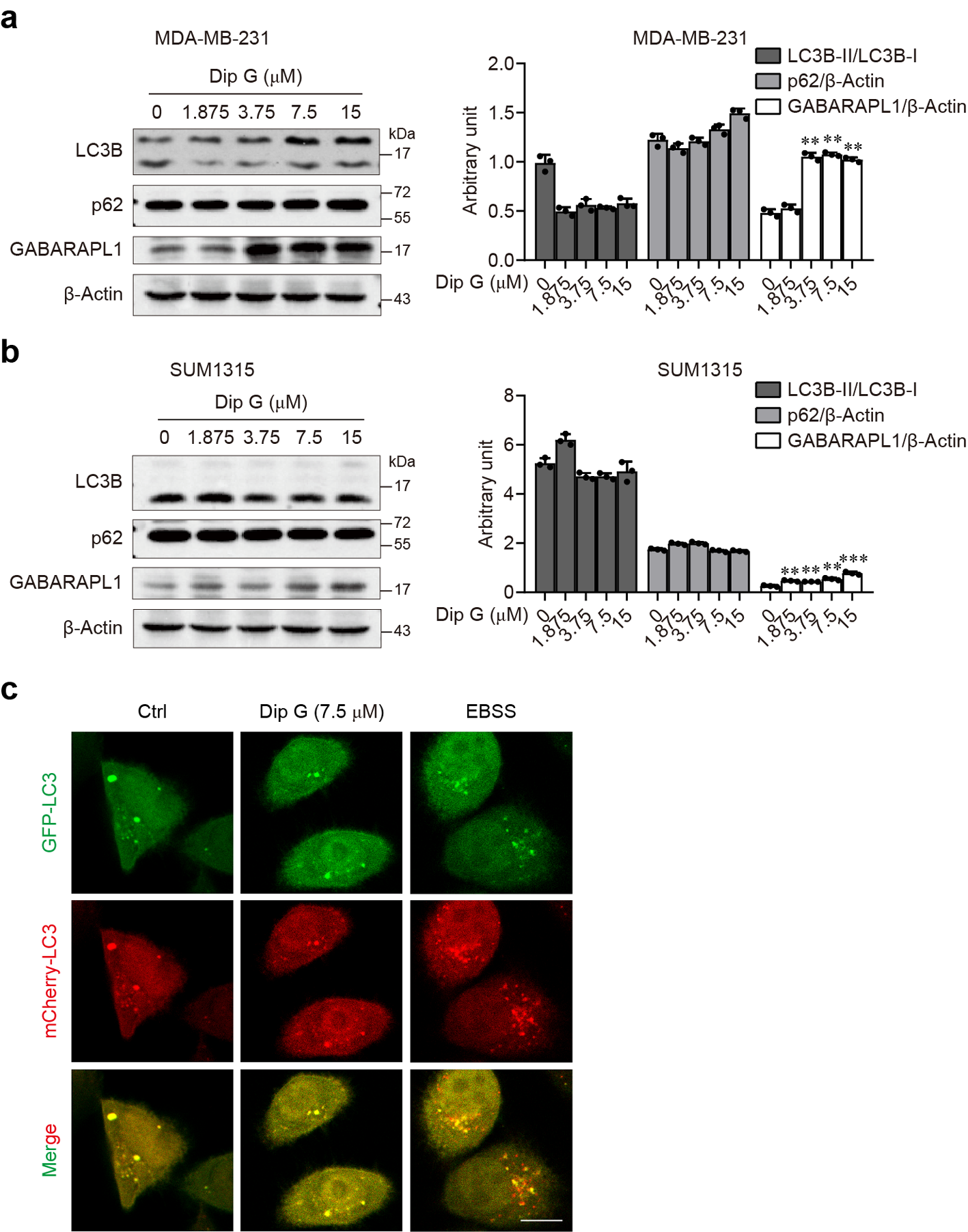

Supplement: Supplementary file 6 — Figure S5 [file 41419_2020_2878_MOESM6_ESM.tif]

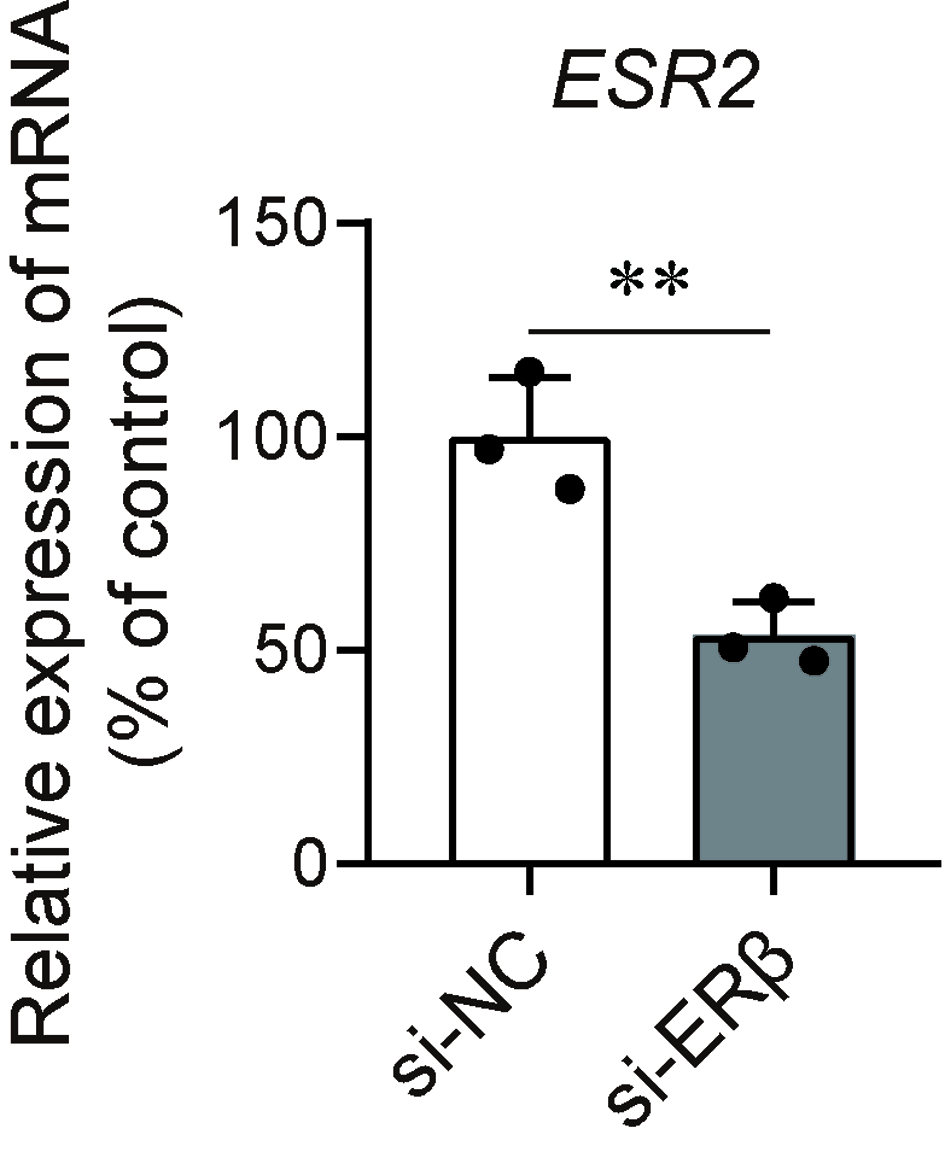

Supplement: Supplementary file 7 — Figure S6 [file 41419_2020_2878_MOESM7_ESM.tif]

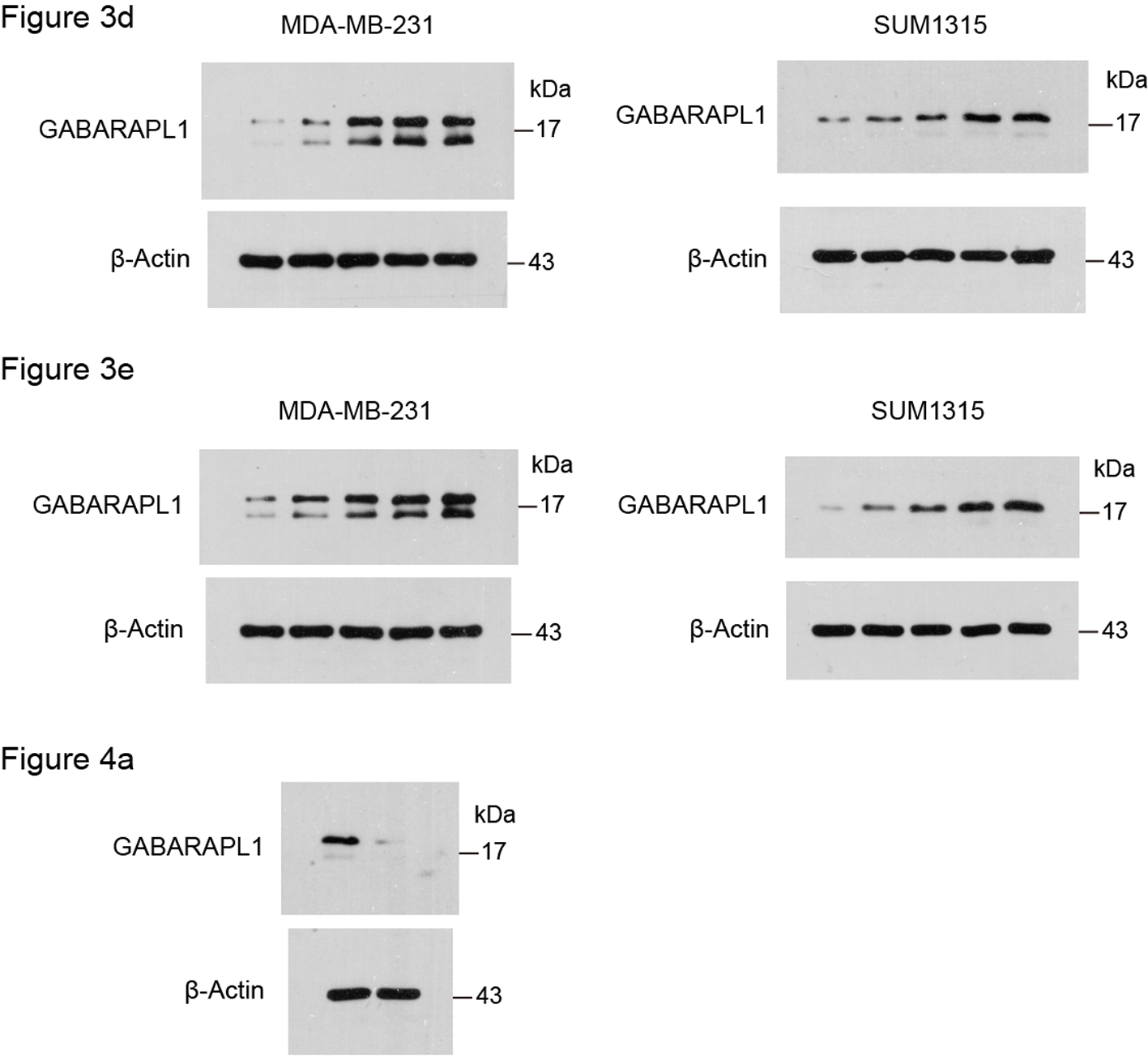

Supplement: Supplementary file 8 — Figure S7 [file 41419_2020_2878_MOESM8_ESM.tif]
