# Supplementary material for: Triggering a switch from basal- to luminal-like breast cancer subtype by the small-molecule diptoindonesin G via induction of GABARAPL1
Source: Cell Death Dis. 2020 Aug 15;11(8):635. doi: 10.1038/s41419-020-02878-z (PMC7429843; doi:10.1038/s41419-020-02878-z)
Supplement: Supplementary file 9 — Supplementary Table legends [file 41419_2020_2878_MOESM9_ESM.docx]

**Supplementary Table Legends**

**Table S1.** **Common** **differentially up-regulated and down-regulated genes between Dip G-treated MDA-MB-231 and HL60 cells.**

**Table S2. Primer sequences for real-time RT-PCR**
