# Supplementary material for: Triggering a switch from basal- to luminal-like breast cancer subtype by the small-molecule diptoindonesin G via induction of GABARAPL1
Source: Cell Death Dis. 2020 Aug 15;11(8):635. doi: 10.1038/s41419-020-02878-z (PMC7429843; doi:10.1038/s41419-020-02878-z)
Supplement: Supplementary file 11 — Table S2 [file 41419_2020_2878_MOESM11_ESM.docx]

**Table S1. Primer sequences for real-time RT-PCR**

| Primer | Forward (5′→3′) | Reverse (5′→3′) |
| --- | --- | --- |
| E-Cadherin | GGCGCCACCTGGAGAGA | TGTCGACCGGTGCAATCTT |
| Vimentin | TACAGGAAGCTGCTGGAAGG | ACCAGAGGGAGTGAATCCAG |
| N-Cadherin | ATCCTACTGGACGGTTCG | TTGGCTAATGGCACTTGA |
| GABARAPL1 | ATGAAGTTCCAGTACAAGGAGGA | GCTTTTGGAGCCTTCTCTACAAT |
| CD44 | CTGCCGCTTTGCAGGTGTA | CATTGTGGGCAAGGTGCTATT |
| CK5 | CCAAGGTTGATGCACTGATGG | TGTCAGAGACATGCGTCTGC |
| FOXA1 | GCAATACTCGCCTTACGGCT | TACACACCTTGGTAGTACGCC |
| GATA3 | CAATGCCTGCGGACTCTACC | GGTGGTGGTCTCGACAGTTCG |
| EpCAM | AATCGTCAATGCCAGTGTACTT | TCTCATCGCAGTCAGGATCATAA |
| ALDH1 | CCGTGGCGTACTATGGATGC | GCAGCAGACGATCTCTTTCGAT |
| BMI1 | GAAATGAAGAGAAGAAGGGA | CCGATCCAATCTGTTCTGGT |
| NANOG | TTTGTGGGCCTGAAGAAAACT | AGGGCTGTCCTGAATAAGCAG |
| ESR1 | GAAAGGTGGGATACGAAAAGACC | GCTGTTCTTCTTAGAGCGTTTGA |
| ESR2 | TCCATCGCCAGTTATCACATCT | CTGGACCAGTAACAGGGCTG |
| ELK3 | ATCTGCTGGACCTCGAACGA | TTCTGCCCGATCACCTTCTTG |
| FBN1 | GCGGAAATCAGTGTATTGTCCC | CAGTGTTGTATGGATCTGGAGC |
| MAP4K4 | GACTCCCCTGCAAAAAGTCTG | GTCCATAGGTGCCATTTCCAA |
| ZEB1 | TTACACCTTTGCATACAGAACCC | TTTACGATTACACCCAGACTGC |
| ERBB3 | GACCCAGGTCTACGATGGGAA | GTGAGCTGAGTCAAGCGGAG |
| XBP1 | CCCTCCAGAACATCTCCCCAT | ACATGACTGGGTCCAAGTTGT |
| TTC39A | CCCTGGACCAGTGCATGAC | TTCTGGGCTTGAGGTAGCTGA |
| SELENBP1 | TCCCCAGTATTGCCAGGTCAT | CGACTTGGTGCTATCACCGAA |
| β-Actin | CATGTACGTTGCTATCCAGGC | CTCCTTAATGTCACGCACGAT |
